# Supplementary material for: Profiles of expression pattern and tissue distribution of host defense peptides genes in different chicken (Gallus gallus) breeds related to body weight
Source: PLoS One. 2020 Dec 28;15(12):e0238675. doi: 10.1371/journal.pone.0238675 (PMC7769465; doi:10.1371/journal.pone.0238675)
Supplement: Attachment — Submitted filename: PLoS One Review Comments.docx [file pone.0238675.s002.docx]

The manuscript by Li, Z., et al. compared the plasma concentrations of several immune parameters (C3, C4, IgA, and IgY) as well as the expression levels of 17 chicken HDP genes in the small intestine, spleen, thymus, and bursa of three different breeds of chickens of varying maturing sizes. A few methodologies are a little confusing and some of the conclusions are not well supported by the data. The following are my specific comments:

1.	Although the authors rightfully indicated that HDP gene expressions are highly tissue-specific, each of 14 chicken beta-defensins and 3 cathelicidins was surprisingly found to be expressed in all four tissues examined including the small intestine, spleen, thymus, and bursa. Please provide the literature support or experimental evidence to show all 17 chicken HDP genes are expressed in each of the four tissue types.

2.	The expression levels of chicken HDP genes was examined in the small intestine. What is the specific segment of the small intestine (duodenum, jejunum or ileum)? For each segment, what is the specific section (proximal, middle, or distal)? These details need to be provided.

3.	In qPCR experiments, a total of 720 samples (= 4 x 60 x 3) were collected from 4 tissue types in each of 60 animals (in 3 breeds) at 3 different ages (weeks 4, 8, and 12). For each sample, qPCR was performed with 17 chicken HDP genes and one reference gene. Each qPCR reaction was said to repeat 3 times based on figure legends. So in the end, a total of 720 x 18 x 3 = 38,880 qPCR reactions were run. Please describe in detail how total RNA were isolated from 720 samples and how 38,880 RT-qPCR reactions were set up, and how data were normalized across hundreds of different PCR plates in order to ensure that the differences in HDP expression levels are not due to technical variations?

4.	Spearman correlation analysis was used to study the correlation between body weight and the expression levels of each of the 17 chicken HDP genes in each of the small intestine, spleen, thymus, and bursa. Please justify why Spearman correlation, not Pearson correlation, was performed. Since HDP expression patterns in three breeds of chickens are significantly different from each other, what is the reason to combine all three breeds? Does analyzing each breed make more sense? Many of the correlation coefficients are nearly 0.65, but surprisingly still insignificant. Please double-check your statistics, particularly given your large sample size (n = 60).

5.	"Host defense peptides" or "HDP" doesn’t need to be italicized in the text because it is not the name of a specific gene. Only a symbol (or abbreviation), not full name, for a gene is italicized. Therefore, "avian beta-defensin 1" or "cathelicidin 2" don’t need to be italicized only when you write *AvBD1* or *Cath2*. Please make corrections in the text.

6.	All figures' resolutions are low and statistics are barely discernable.

7.	Move all tables and figure legends to the end of the text.

8.	Grammatical errors are scattered through the text and need to be corrected.

9.	Lines 24-25 - "Selection for increased body weight is suggested to be related to reduced immune response". Please provide literature support.

10. Lines 32-34 – "These results indicated that the HDP immune regulatory roles in small intestine acted as first line of defense in innate immunity in local breeds, and as an adaptive immunity in broiler chickens". I don’t understand the statement. Please explain how it is supported by the experimental results?

11. Line 35 – "Selection was associated with the expression profiles of HDP genes in a chicken, age, and organ-specific manner". Change it to "Selection was associated with different expression expressions of HDP genes in breed-, age-, and organ-specific manners".
