# Supplementary material for: Profiles of expression pattern and tissue distribution of host defense peptides genes in different chicken (Gallus gallus) breeds related to body weight
Source: PLoS One. 2020 Dec 28;15(12):e0238675. doi: 10.1371/journal.pone.0238675 (PMC7769465; doi:10.1371/journal.pone.0238675)
Supplement: Attachment — Submitted filename: Rebuttal letter.docx [file pone.0238675.s003.docx]

Dear Dr. Kogut,

Thank you for the possibility to submit a revised manuscript. Please find attached a revised manuscript. Please find below a detailed listing of all changes made to the manuscript. We herewith submit the revised manuscript to PLOS ONE.

Best Regards,

Marinus te Pas

Corresponding author

1. Please ensure that your manuscript meets PLOS ONE's style requirements, including those for file naming. The PLOS ONE style templates can be found at

<https://journals.plos.org/plosone/s/file?id=wjVg/PLOSOne_formatting_sample_main_body.pdf>

and

<https://journals.plos.org/plosone/s/file?id=ba62/PLOSOne_formatting_sample_title_authors_affiliations.pdf>

**We have changed the format of the text as required. The headers were formatted (bold + 18 / 16 pt,, no italics anymore) before the track changes was on because this gave too many remarks in the document and affected readability. We hope that the editor can accept this.**

2. We note that you have included the phrase “data not shown” in your manuscript. Unfortunately, this does not meet our data sharing requirements. PLOS does not permit references to inaccessible data. We require that authors provide all relevant data within the paper, Supporting Information files, or in an acceptable, public repository. Please add a citation to support this phrase or upload the data that corresponds with these findings to a stable repository (such as Figshare or Dryad) and provide and URLs, DOIs, or accession numbers that may be used to access these data. Or, if the data are not a core part of the research being presented in your study, we ask that you remove the phrase that refers to these data. **This data was just a check for the laboratory methods indicating that the results are reliable. We have deleted the text.**

4. We note that you have indicated that data from this study are available upon request. PLOS only allows data to be available upon request if there are legal or ethical restrictions on sharing data publicly. For more information on unacceptable data access restrictions, please see http://journals.plos.org/plosone/s/data-availability#loc-unacceptable-data-access-restrictions. **There are no restrictions on data availability. Our problem was that we included all data in the manuscript, so there are no additional data. Just in case someone would be interested in the raw data or share samples we are offering the possibility for contact to discuss the (technical) possibilities. Please contact Prof. Jia.**

In your revised cover letter, please address the following prompts:

a) If there are ethical or legal restrictions on sharing a de-identified data set, please explain them in detail (e.g., data contain potentially sensitive information, data are owned by a third-party organization, etc.) and who has imposed them (e.g., an ethics committee). Please also provide contact information for a data access committee, ethics committee, or other institutional body to which data requests may be sent. **There are no restrictions. See also what we explained above (point 4).**

b) If there are no restrictions, please upload the minimal anonymized data set necessary to replicate your study findings as either Supporting Information files or to a stable, public repository and provide us with the relevant URLs, DOIs, or accession numbers. For a list of acceptable repositories, please see http://journals.plos.org/plosone/s/data-availability#loc-recommended-repositories. **Please note that these data are very different from omics data such as one can find in several databases. These data are probably of less value for the readers. And please note also that if readers contact us they will be supplied with the data and help for analyzing and interpretation. The reviewers also noted this: see their answers to question 3 below, where they stated that all data underlying the findings in the manuscript are made fully available. However, we have also no objection against providing all raw data. Therefore, we have included all raw data in a supporting data file. On top of this we like to keep our offer for contact and support.**

We will update your Data Availability statement on your behalf to reflect the information you provide. **Thank you!**

Reviewers' comments:

Reviewer's Responses to Questions

Comments to the Author

1. Is the manuscript technically sound, and do the data support the conclusions?

The manuscript must describe a technically sound piece of scientific research with data that supports the conclusions. Experiments must have been conducted rigorously, with appropriate controls, replication, and sample sizes. The conclusions must be drawn appropriately based on the data presented.

Reviewer #1: Yes

Reviewer #2: Yes **Thank you.**

________________________________________

2. Has the statistical analysis been performed appropriately and rigorously?

Reviewer #1: No **We have updated our description. We hope that the reviewer is satisfied with this.**

Reviewer #2: I Don't Know

________________________________________

3. Have the authors made all data underlying the findings in their manuscript fully available?

The PLOS Data policy requires authors to make all data underlying the findings described in their manuscript fully available without restriction, with rare exception (please refer to the Data Availability Statement in the manuscript PDF file). The data should be provided as part of the manuscript or its supporting information, or deposited to a public repository. For example, in addition to summary statistics, the data points behind means, medians and variance measures should be available. If there are restrictions on publicly sharing data—e.g. participant privacy or use of data from a third party—those must be specified.

Reviewer #1: Yes

Reviewer #2: Yes **Thank you!**

________________________________________

4. Is the manuscript presented in an intelligible fashion and written in standard English?

PLOS ONE does not copyedit accepted manuscripts, so the language in submitted articles must be clear, correct, and unambiguous. Any typographical or grammatical errors should be corrected at revision, so please note any specific errors here.

Reviewer #1: No

Reviewer #2: No **We have improved the text of the manuscript and we hope that the reviewers can accept the present draft of the manuscript.**

________________________________________

5. Review Comments to the Author

Reviewer #1: The manuscript by Li, Z., et al. compared the plasma concentrations of several immune parameters (C3, C4, IgA, and IgY) as well as the expression levels of 17 chicken HDP genes in the small intestine, spleen, thymus, and bursa of three different breeds of chickens of varying maturing sizes. A few methodologies are a little confusing and some of the conclusions are not well supported by the data. **We hope that the revised draft clarifies this.** The following are my specific comments:

1. Although the authors rightfully indicated that HDP gene expressions are highly tissue-specific, each of 14 chicken beta-defensins and 3 cathelicidins was surprisingly found to be expressed in all four tissues examined including the small intestine, spleen, thymus, and bursa. Please provide the literature support or experimental evidence to show all 17 chicken HDP genes are expressed in each of the four tissue types. **The HDP genes are expressed very widely throughout the body. We provide evidence with the papers of Yacoub et al., 2006; Van Dijk et al., 2008, 2011; Achanta et al., 2012; Xu et al., 2015. We did add these papers as reference numbers 11-15. Together these papers show a wide pattern of expression in many tissues – including our tissues – as we observed too. This information was included in the Introduction, and the references were inserted in the Reference list after the former reference number 10. The reference numbering was adjusted to the new references.**

2. The expression levels of chicken HDP genes was examined in the small intestine. What is the specific segment of the small intestine (duodenum, jejunum or ileum)? For each segment, what is the specific section (proximal, middle, or distal)? These details need to be provided. **We have added: The small intestine was sampled in the mid duodenum.**

3. In qPCR experiments, a total of 720 samples (= 4 x 60 x 3) were collected from 4 tissue types in each of 60 animals (in 3 breeds) at 3 different ages (weeks 4, 8, and 12). For each sample, qPCR was performed with 17 chicken HDP genes and one reference gene. Each qPCR reaction was said to repeat 3 times based on figure legends. So in the end, a total of 720 x 18 x 3 = 38,880 qPCR reactions were run. Please describe in detail how total RNA were isolated from 720 samples and how 38,880 RT-qPCR reactions were set up, and how data were normalized across hundreds of different PCR plates in order to ensure that the differences in HDP expression levels are not due to technical variations? **This is indeed correct – many of the authors worked on it. Some details: Three PhD students including Zhengtian Li, Irfan Ahmed and Zhiqiang Xu together with other several master students performed this work. Fufthermore, some of data in this manuscript were from thesis of two master students (Wenyuan Hu and Ziqing Jiang). – Please note that these theses are in Chinese and not generally available. Therefore, we feel that it is not valuable to give reference to these theses. Note: we feel that this information is valuable for reviewers to judge the work, but it is less valuable for readers. Therefore, we did not include this into the manuscript. However, if the reviewer likes it we are willing to add this information to the Materials and Methods section.**

4. Spearman correlation analysis was used to study the correlation between body weight and the expression levels of each of the 17 chicken HDP genes in each of the small intestine, spleen, thymus, and bursa. Please justify why Spearman correlation, not Pearson correlation, was performed. **Spearman** **correlation is a non-parametric statistical method and has a wide range of application, which does not require the distribution of original variables.** **In our study, the correlations between the body weight (BW) and mRNA expression of HDPs in different organs were analyzed, while the body weight (BW) and mRNA expression of HDPs were from three chicken breed and different ages (4, 8 and 12 weeks-old), and the original data nonconformed the normal distribution. So Spearman correlation was used. We have added this argument to the Materials and Methods section: Spearman correlation was used instead of Pearson correlation because Spearman correlation is a non-parametric statistical method and has a wide range of application, which does not require the distribution of original variables. The correlations between the body weight (BW) and mRNA expression of HDPs in different organs were analyzed, while the body weight (BW) and mRNA expression of HDPs were from three chicken breed and different ages (4, 8 and 12 weeks-old), and the original data were not normal distributed.** Since HDP expression patterns in three breeds of chickens are significantly different from each other, what is the reason to combine all three breeds? **The purpose of the study is to analyze the expression of a number of genes regulating the immune capacity in chicken with a breeding background varying from no selection at all to heavily breed for increased body weight. Thus, it makes more sense to compare between the breeds too. We agree with the reviewer that this should be added to the manuscript. We added: We analyzed all breeds together to compare the chicken varying from no selection to heavily selected for body weight.** Does analyzing each breed make more sense? **It can be done, but it will not provide more information.** Many of the correlation coefficients are nearly 0.65, but surprisingly still insignificant. Please double-check your statistics, particularly given your large sample size (n = 60). **This is indeed surprising (and unfortunate), but we have to accept this fact.**

5. "Host defense peptides" or "HDP" doesn’t need to be italicized in the text because it is not the name of a specific gene. Only a symbol (or abbreviation), not full name, for a gene is italicized. Therefore, "avian beta-defensin 1" or "cathelicidin 2" don’t need to be italicized only when you write AvBD1 or Cath2. Please make corrections in the text. **We have corrected it throughout the entire manuscript. There are too many places to mention each of them here specifically.**

6. All figures' resolutions are low and statistics are barely discernable. **We have provided new Figures.**

7. Move all tables and figure legends to the end of the text. **We can understand this comment, however, this is the format of PLOS ONE. So, we cannot change this.**

8. Grammatical errors are scattered through the text and need to be corrected. **We have improved the text, and we hope that the reviewer is satisfied now. See also changes made according to the comments below.**

9. Lines 24-25 - "Selection for increased body weight is suggested to be related to reduced immune response". Please provide literature support. **This is indeed not very clear. In the Introduction we specified: “As a consequence of prolonged selection for enhanced body weight and growth rate broilers face reduced immune functioning [1-3]. However, it is feasible to breed broilers for enhanced innate immune reactivity [4]. Therefore, a relation between body weight and regulation of immune functions was expected.” Thus, this was our hypothesis. Therefore, we have changed the text in the Abstract: Selection for increased body weight is hypothesized to be related to reduced immune response.**

10. Lines 32-34 – "These results indicated that the HDP immune regulatory roles in small intestine acted as first line of defense in innate immunity in local breeds, and as an adaptive immunity in broiler chickens". I don’t understand the statement. Please explain how it is supported by the experimental results? **Our previous results indicated that the immune indexes of Daweishan mini chicken was higher than other chickens. These are non-selected chickens. This suggests that selection (for body weight) relates to immune capacity, as indicated in our hypothesis (see the response to comment 9). The problem is that these lines are in the abstract, so little space is available. To indicate that this relates to our results we have changed “These results” into “Our results”. We hope that the reviewer can agree on this.**

11. Line 35 – "Selection was associated with the expression profiles of HDP genes in a chicken, age, and organ-specific manner". Change it to "Selection was associated with different expression expressions of HDP genes in breed-, age-, and organ-specific manners". **Done**

Reviewer #2: Lines 45 – 57: It is important to differentiate between HDP genes and the peptides for which they encode. As these lines seem to be referring to the molecules rather than the genes, the phrase “HDP genes” should be replaced with “HDPs” or “HDP peptides” **The reviewer is right. We have specified two times changing genes into peptides.**

Lines 78 – 79: “20 chickens from each chickens” is confusing. Should be changed to “20 chickens from each line” **Done**

Line 105: Reads “jugular of wing”. I believe it should be changed to “jugular or wing” **Done**

Lines 134 – 135: To my knowledge, repeated t-test is never an appropriate analysis. ANOVA should be used in all situations that compare more than 2 groups. If you keep the repeated t-test please clarify in which situations it was used. **The reviewer is right. The text as it stand is indeed very confusing. The meaning was: we used ANOVA to compare among the three breed and within breeds more than 2 groups - we just used the t-test to confirm the significance between the two age within breed. We have deleted the text.**

Lines 145 – 147: Seems to be some discrepancy between text and the figure labels regarding which time points are significant between breeds. For example, text says “There was no significant difference in the serum complement C4 and IgA concentrations at 8 – 12 weeks” but figure indicates IgA significance at week 8. Please double check figures and clarify. **We have done so and found that the reviewer is right. We have deleted the sentence. The manuscript is much clearer without the sentence.**

Line 196: Wording “mRNA expression” is a repeat. Please remove. **Done**

Line 196 – 197: This seems to be a repeat of lines 188 to 190. In general, the two paragraphs in this section would make more sense if combined into one. **Done**

Line 210: Change “exception” to “except” **Done**

Line 209 – 214: Please clarify to which week each sentence is referring rather than just including the panels in parenthesis. **Done**

Line 219 – 220: This sentence does not make sense. Please revise. **This sentence was indeed very unclear. We changed the sentence into: In the thymus of the Daweishan mini chicken an age dependent decreased expression was observed in AvBD genes except for AvBD 12 and 14.**

Overall resolution and quality of the figures needs to be improved. They are all fuzzy making the wording and significance indicators difficult to read. Also, please indicate the statistical test used in each situation in the figure legends. **We provide new Figures.**

While revising your submission, please upload your figure files to the Preflight Analysis and Conversion Engine (PACE) digital diagnostic tool, https://pacev2.apexcovantage.com/. PACE helps ensure that figures meet PLOS requirements. To use PACE, you must first register as a user. Registration is free. Then, login and navigate to the UPLOAD tab, where you will find detailed instructions on how to use the tool. If you encounter any issues or have any questions when using PACE, please email PLOS at figures@plos.org. Please note that Supporting Information files do not need this step. **Done. We will upload the PACE revised files.**
